# Testosterone Impairs Endometrial Receptivity Markers and Trophoblast Spheroid Attachment *In Vitro*: Reversal by Flutamide in a Model of Polyendocrine Metabolic Ovarian Syndrome

**DOI:** 10.3390/cimb48101090

**Published:** 2026-10-21

**Authors:** Claire V. Harper, Beth J. Reeves, Chloe S. Elliott, Raabiah Sidat, Jayne C. Charnock

**Affiliations:** Department of Biology, Edge Hill University, Ormskirk L39 4QP, UK; bethreeves8@icloud.com (B.J.R.); elliottch@edgehill.ac.uk (C.S.E.); sidatr@edgehill.ac.uk (R.S.)

**Keywords:** polyendocrine metabolic ovarian syndrome, polycystic ovary syndrome, testosterone, embryo attachment, gene expression, flutamide, androgen

## Abstract

Polyendocrine metabolic ovarian syndrome (PMOS; previously termed polycystic ovary syndrome, PCOS) is a multi-faceted reproductive and endocrinological condition that affects approximately 10% of women of reproductive age. It is typically characterised by chronic anovulation and elevated levels of the androgen testosterone, which have been linked to reduced implantation rates and fertility outcomes. In this study, we investigated whether increased testosterone treatment affected attachment of trophoblast spheroids to an endometrial cell monolayer and the expression of genes known to be involved in the attachment process. Attachment of BeWo spheroids to Ishikawa endometrial cells was significantly reduced when Ishikawa cells were pre-treated for 24 h with pathological and suprapathological doses of testosterone compared to the results for the untreated control, with no significant effect measured following treatment with a physiological testosterone dose (1 nM). Expression of the transcription factor gene *HOXA10*; integrin *β3* (a component of the αVβ3 molecule) and *SPP1* (the gene expressing the ligand osteopontin), were significantly decreased in Ishikawa cells following treatment with 10 nM testosterone, along with αVβ3 protein. Treatment with the nonsteroidal antiandrogen flutamide (1 µM) rescued the testosterone-induced decrease in BeWo spheroid–endometrial cell attachment and associated decreases in gene expression. These results suggest that increased levels of testosterone decrease endometrial receptivity and downstream embryo attachment through negatively affecting the expression of genes required for the success of these processes. This testosterone-induced decrease can be reversed in vitro with flutamide treatment. These results highlight the importance of considering endometrial receptivity in the treatment of PMOS-induced infertility.

## 1. Introduction

Polyendocrine metabolic ovarian syndrome (PMOS; previously referred to as polycystic ovary syndrome) is a reproductive and endocrinological disorder that affects 10–13% of women of reproductive age [1]. PMOS is recognised as one of the most common endocrine disorders present in women and presents with several main characteristics: polycystic ovaries, oligo-/amenorrhea due to oligo-/anovulation, elevated circulating concentrations of androgens and/or signs of hyperandrogenism, and insulin resistance [2,3]. One of the main concerns that women with PMOS face is infertility, and PMOS is responsible for 70–80% of anovulatory infertility cases, making it the most frequent cause [4,5]. However, inducing ovulation in women with PMOS does not lead to increased endometrial receptivity. This is best illustrated in fertility treatment programmes such as in vitro fertilization (IVF), for which women with PMOS show lower success rates, even when high-quality embryos are being transferred [6]. Endometrial receptivity is vital for embryo implantation, and the low success rates observed indicate that anovulation is not the only cause of infertility in women with PMOS [7,8]. Altered expression of adhesion molecules within the endometrium appears to be a significant factor the in reduction of receptivity in PMOS women [9].

In humans, the early implantation of the blastocyst is essential for a successful pregnancy and is vital for the formation of the foetal–maternal interface. Attachment of the embryo involves the physical and physiological contact of the trophectoderm of the blastocyst with the uterine luminal epithelium. For pregnancy to be successful, the embryo must attach and implant within a given ‘window of implantation’ (WOI) during which the endometrium is receptive [10,11]. Integrins are cell adhesion molecules that are considered to be good indicators of endometrial receptivity and are essential for cell–cell contact. During embryo implantation, multiple integrins, one of which is αVβ3, play a role in making implantation a success. αVβ3 is comprised of alpha-V and beta-3 subunits [12]. Throughout the menstrual cycle, the production of αVβ3 varies—in the embryo-receptive mid-secretory phase, αVβ3 production is increased within the endometrium, and the β3 subunit, in particular, increases in expression during the WOI [13].

In women for whom reduced endometrial receptivity is the reason for their infertility, a reduction in αVβ3 integrin is measured [14,15,16,17]. There are extensive reports of a similar reduction in human PMOS endometrium during the secretory phase [2], and various investigations of the endometrium of mice with induced PMOS have also confirmed that the expression of the αVβ3 subunits (αV and β3) is significantly decreased [17,18]. Osteopontin (OPN), a secreted protein ligand of αVβ3 that is upregulated in receptive endometria, plays known roles in embryo adhesion and trophoblast–endometrial cell interactions at the uterine–placental interface [19,20,21]. Differential regulation of OPN in infertile PMOS patients has been reported [22,23]. Testosterone is known to suppress OPN expression within the renal system; however, its impact on uterine OPN is unknown [24].

*HOX* genes are required for the transcriptional regulation of endometrial development and receptivity. The HOXA10 transcription factor is essential for the development of the uterus during organogenesis, as well as for further development of the endometrium in adults, enabling uterine receptivity for embryo implantation [25]. The role of HOXA10 in uterine development is illustrated in female mice with a targeted disruption of HOXA10. These female mice present with infertility due to reduced endometrial receptivity, preventing embryo implantation from successfully taking place [26]. HOXA10 is also known to be expressed at the time of embryo implantation in the endometrium [27]. A relationship between HOXA10 and the β3 integrin subunit of αVβ3 has been determined, whereby when HOXA10 expression is significantly reduced, the *β3* subunit expression is also reduced [28]. Cermik et al. [25] previously found that testosterone treatment of endometrial cells decreases HOXA10 expression by approximately 50% of the control values. This finding, along with the decreased HOXA10 levels seen in the endometrium of patients with PMOS, suggest that testosterone may impact HOXA10 expression in the endometrium of women with PMOS [25,28,29].

Conversely, flutamide, an androgen receptor antagonist, can be used to treat hyperandrogenism in women with PMOS. De Leo et al. [30] illustrated a reduction in androgen synthesis in PMOS patients, alongside a restoration of ovulatory cycles and a reduction in ovarian size and follicle number following flutamide treatment. Additionally, flutamide has been shown to restore the observed testosterone-driven decrease in HOXA10 expression in vitro [25].

In this study, the impact of testosterone on embryo attachment using an in vitro trophoblast spheroid model has been investigated, along with changes to key attachment proteins and their regulatory genes. In addition, the effect of flutamide on this process, as a means of rescuing attachment and elucidating the underlying mechanism of testosterone action on attachment, was determined.

## 2. Methods

### 2.1. Cell Culture

Human Ishikawa endometrial adenocarcinoma cells (ECACC, UK, 99040201) and BeWo choriocarcinoma cells (ECACC, UK, 86082803) were maintained in DMEM/F12 (Gibco, (Grand Island, NY, USA) supplemented with 10% foetal bovine serum (FBS, **), 1% (2 mM) L-glutamine (Thermo Fisher Scientific, Waltham, MA, USA) and incubated at 37 °C, 5% CO_2_. Regular mycoplasma testing was performed. All experiments used cells between passage 4 and 26. The media was replaced with serum-free DMEM/F-12 and incubated overnight prior to treatments.

### 2.2. Cell Treatments

Ishikawa cells were treated with a range of testosterone doses (1 nM, 10 nM, and 100 nM; Sigma, (St. Louis, MO, USA), flutamide (1 µM; Sigma, (St. Louis, MO, USA), combined flutamide and testosterone, or the equivalent methanol control and incubated for 24 h at 37 °C, 5% CO_2_. Images were captured using a Leica DMi8 inverted microscope (Leica Microsystems, Wetzlar, Germany) using a 10× objective to determine morphological alterations following testosterone treatment.

### 2.3. Cell Viability Assay

Ishikawa cells were plated in a 24-well plate (4 × 10^4^ cells per well in 1 mL), incubated overnight, and then treated with testosterone or methanol control for 24 h, as described above. A Prestoblue assay was performed, following the manufacturer’s instructions (Thermo Fisher Scientific, Waltham, MA, USA). The fluorescent signal was measured using a BMG Fluostar Omega Microplate reader at a wavelength of 570 nm, with a reference wavelength set at 600 nm.

### 2.4. Trophoblast Spheroid Generation

BeWo trophoblast spheroids were generated following a modified version of the method by Ho et al. [31]. Briefly, flasks were coated with 10% Polyvinylpyrrolidone (PVP, Thermo Fisher Scientific, Waltham, MA, USA) and incubated at room temperature for 30 min. Following removal of the PVP, flasks were washed with PBS, and 2 × 10^6^ BeWo cells were added to the coated flask, followed by incubation at 37 °C with 5% CO_2_ on an orbital shaker at 60 RPM for 4 h, followed by static incubation for 24 h.

### 2.5. Trophoblast Spheroid Attachment Assay

BeWo spheroids were isolated by filtration through a 100 µm cell filter. Small cell clumps were removed through a 40 µm cell filter. Spheroids collected on the filter were washed with 2.5 mL of media. A total of 100 µL of BeWo spheroid solution was added to treatment media of each well of the Ishikawa cells and incubated for 2 h at 37 °C, 5% CO_2_. The number of spheroids per well was counted by eye using a Leica DMI8 inverted microscope (Leica Microsystems, Wetzlar, Germany) and recounted following two washes with 1 mL of PBS. Four replicate wells were counted for each treatment in each experiment, resulting in 10 to 130 spheroids per well. Spheroids were considered attached if they remained present following the PBS washes.

The percentage of attachment was calculated as the [number of attached spheroids (post-PBS washes)/number of total spheroids (pre-PBS washes)] × 100%.

CellTracker Red CMTPX dye (Thermo Fisher Scientific, Waltham, MA, USA) was used to stain the BeWo spheroids, following manufacturer’s instructions. Images were captured using a Leica DMi8 fluorescent inverted microscope (Leica Microsystems, Wetzlar, Germany) and processed using Leica applications suite X 3.7.5.

### 2.6. Gene Expression Analysis

Ishikawa cells were plated at a density of 3 × 10^5^ cells per 60 mm dish and incubated for 24 h. Following treatment, cell lysates were homogenised (Qiashredder, Qiagen, Takara, Otsu, Japan), and total RNA was extracted using an RNeasy kit (Qiagen), according to manufacturer’s instructions. RNA concentration was determined using a Nanodrop ND-1000 Spectrophotometer (Thermo Fisher Scientific, Waltham, MA, USA), and 1 µg RNA was reverse-transcribed and amplified using a High-Capacity RNA-cDNA kit (Thermo Fisher Scientific, Waltham, MA, USA). For RT-qPCR, cDNA was diluted to 1:20 with RNase-free water and amplified using PowerUp SYBR green MasterMix (Thermo Fisher Scientific, Waltham, MA, USA). Relative quantification of gene expression was conducted using the *β-actin* expression level as an endogenous control. Primer sequences used were *HOXA10* F: ctgaggtcaatggtgcaaagga, R: tttgccaacctgcatgtcca (taken from Matsuzaki, Canis [32]); *β3* F: gagttcccagtgagtgaggc, R: cttgagtgacctgggagctg; *SPP1* F: acctcacacatggaaagcga, R: caatcagaaggcgcgttcag; *β-actin* F: catccgcaaagacctgtacg, R: tctccttctgcatcctgtcg. Gene expression was determined using a QuantStudio 5 Real-time PCR system (Applied Biosystems, Warrington, UK).

### 2.7. Immunocytochemistry

Ishikawa cells were plated onto glass coverslips (2.5 × 10^5^) in 60 mm dishes and incubated overnight at 37 °C, 5% CO_2_. Cells were fixed for 6 min using 4% paraformaldehyde, permeabilized in 0.1% Triton X-100 and then blocked in 1% BSA/PBS. Coverslips were then incubated with αvβ3 anti-mouse antibody (Abcam, Shanghai, China, ab78289) overnight at 4 °C, followed by incubation with goat anti-mouse IgG-Alexa Fluor 488 (Abcam, Shanghai, China, ab150113) for 1 h at room temperature. Nuclear DNA was labelled with DAPI (Vectashield, Merck, Darmstadt, Germany), and coverslips were imaged at 20× magnification on a Leica DMI8 inverted microscope (Leica Microsystems, Wetzlar, Germany). A negative staining control was used to set the level of background cellular autofluorescence. Mean fluorescence intensity from each individual cell was quantified using ImageJ/Fiji 1.54p.

### 2.8. Data Analysis

Data were collated in Microsoft Excel and analysed in GraphPad Prism (version 11.1.0). At least three independent replicate experiments were performed per analysis. ANOVA or Friedman tests were used with post hoc multiple comparisons tests for grouped analyses following tests for normality using a Shapiro–Wilk test. Data were paired across treatments. Statistical significance was determined as *p* < 0.05.

## 3. Results

### 3.1. Effect of Testosterone on Ishikawa Cell Viability and Morphology

**Figure 1:**
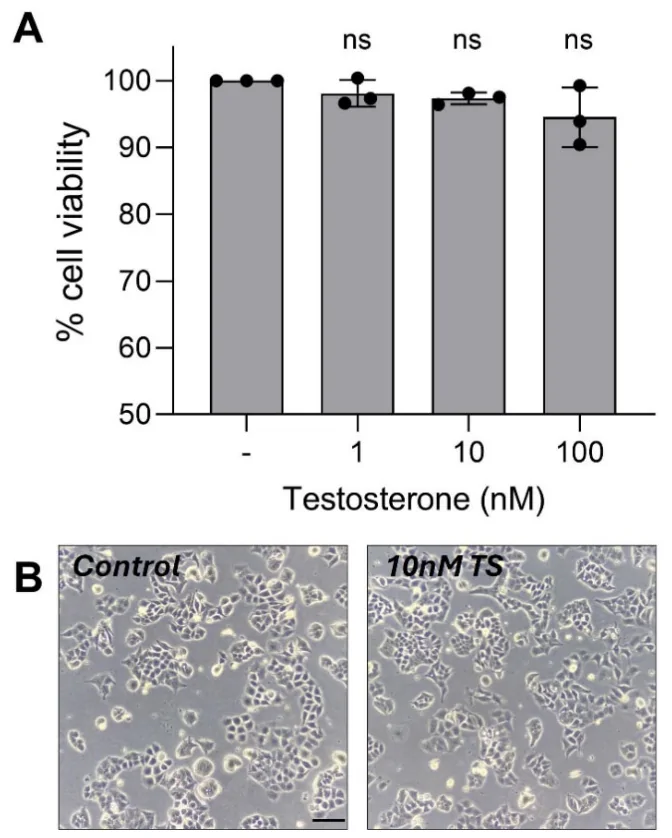
Cell viability measurement in Ishikawa cells following testosterone treatment. (**A**) Ishikawa cells treated with 1 nM, 10 nM, 100 nM testosterone (TS) or vehicle control for 24 h were assessed for change in cell viability using a PrestoBlue assay. Bars show mean +/− standard deviation with individual experimental data shown. ns = not significant, ANOVA with post hoc Dunnett’s multiple comparisons test, *n* = 3 experiments. (**B**) Ishikawa cell images following 24 h treatment with vehicle and 10 nM testosterone. Scale bar shows 100 µm.

To determine whether 24 h testosterone treatment affected the viability of Ishikawa endometrial cells, cell images were captured and a PrestoBlue assay was performed. There was no significant effect on cell viability across the dose range 1–100 nM of testosterone (*p* = 0.25, *n =* 3 experiments; Figure 1A). Cell morphology was maintained following testosterone treatment (Figure 1B).

### 3.2. Testosterone Treatment Impairs Model Embryo–Endometrial Attachment

**Figure 2:**
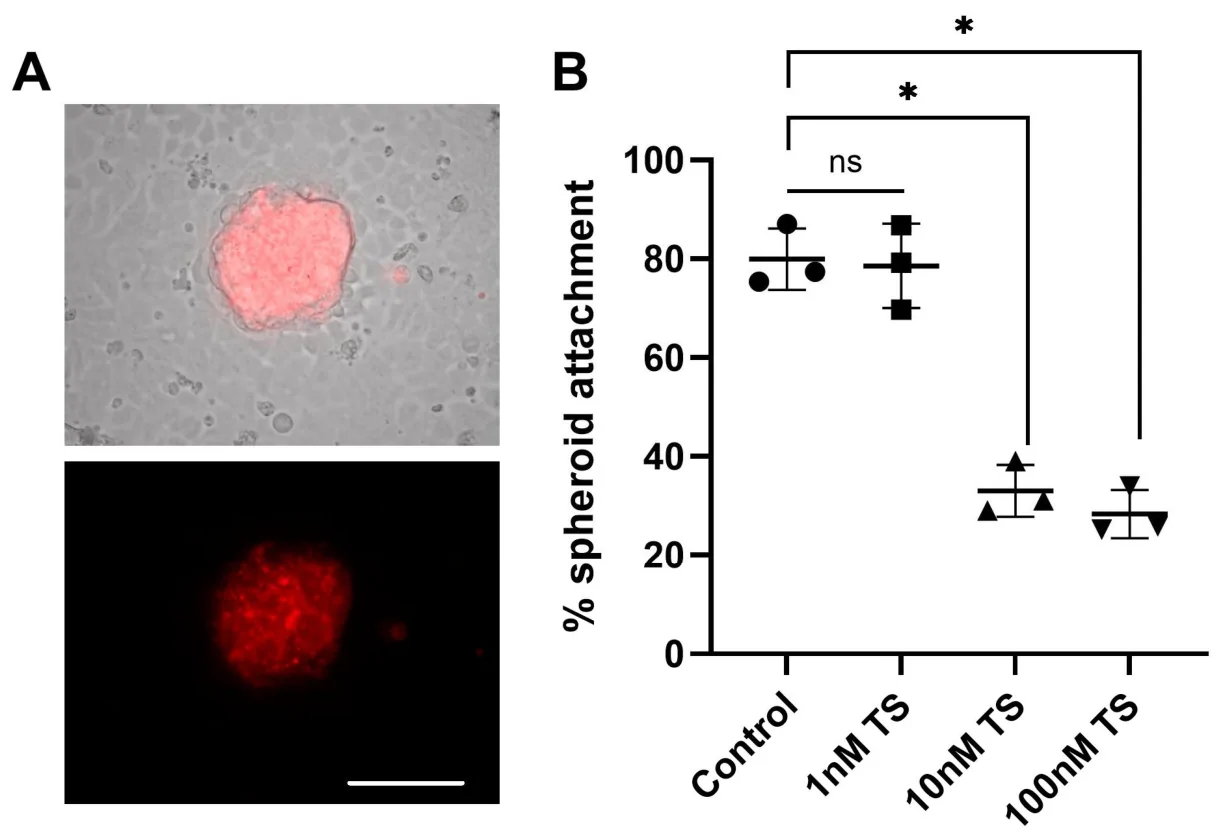
BeWo spheroid attachment assay. (**A**) Attachment of a fluorescently labelled BeWo spheroid to an Ishikawa cell monolayer using CellTracker Red CMTPX dye. Scale bar represents 100 µm. (**B**) Percentage of spheroid attachment to Ishikawa cells pre-treated with 1 nM, 10 nM, 100 nM testosterone (TS) or vehicle control for 24 h (ns = not significant; * = *p* < 0.05; ANOVA with post hoc Dunnett’s multiple comparisons test; *n* = 3 experiments). Bars show mean +/− standard deviation with individual experimental data shown.

BeWo trophoblast spheroids were used as a model of the human embryo to determine whether testosterone pre-treatment modulated the attachment to an Ishikawa cell monolayer (Figure 2A). The 24 h testosterone treatment caused a threshold effect of inhibition of model embryos to the endometrial cell layer, with significant inhibition in attachment occurring at testosterone doses of 10 nM and 100 nM (*p* = 0.029 and *p* = 0.019, respectively, *n* = 3 experiments; Figure 2B). Model embryo–endometrial attachment at these testosterone doses was decreased by over 50% when compared to the attachment rates for the control Ishikawa cells (Figure 2B). No significant effect on model embryo–endometrial cell attachment was measured following treatment of the Ishikawa cells with 1 nM testosterone for 24 h (*p* > 0.98, *n* = 3 experiments; Figure 2B).

### 3.3. Testosterone Inhibits Expression of Endometrial Receptivity Markers

**Figure 3:**
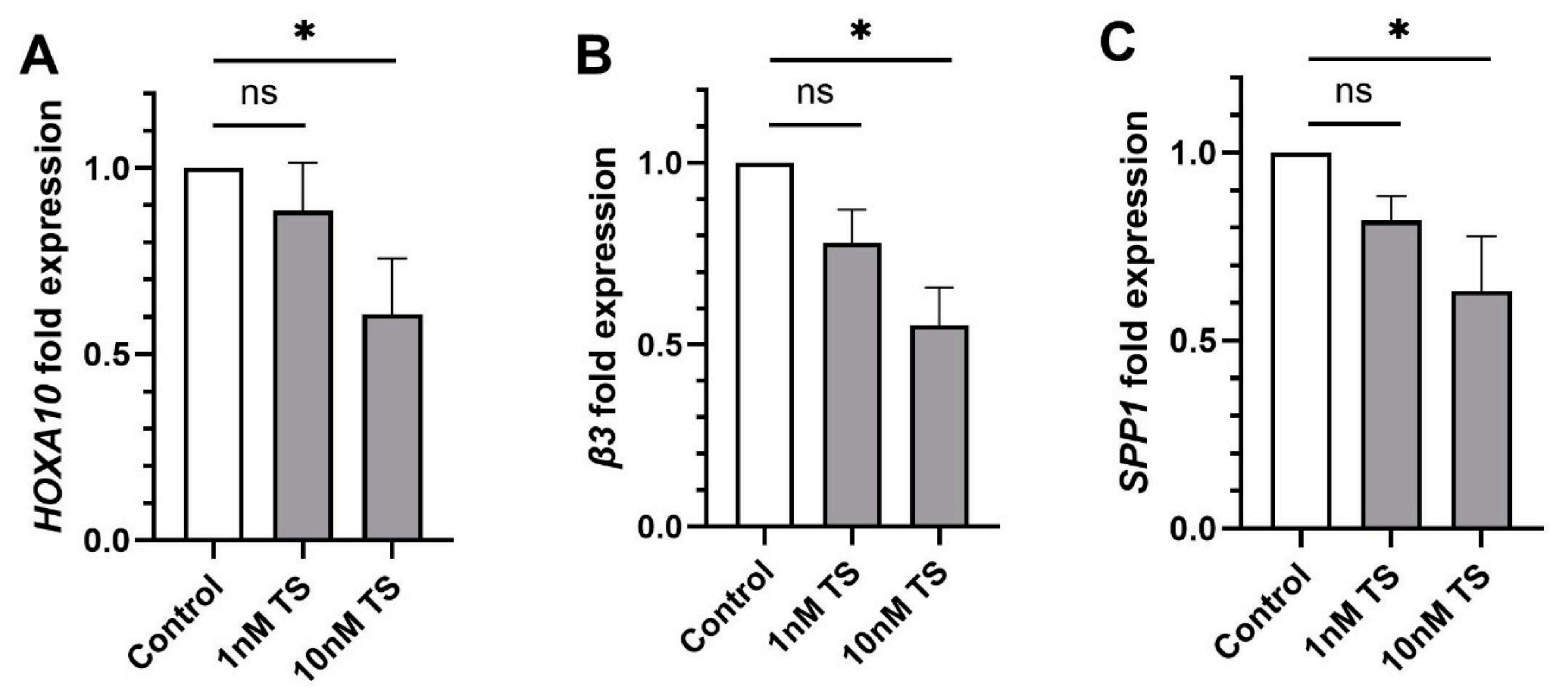
Gene expression of the transcription factor *HOXA10* (**A**), *β3* integrin that forms part of the vitronectin receptor αVβ3 (**B**), and the osteopontin gene *SPP1* (**C**) in Ishikawa cells treated with 1 nM and 10 nM of testosterone (TS) and vehicle control for 24 h. Graphs show mean +/− SD. ns = not significant. * = *p* < 0.05; ANOVA with post hoc Dunnett’s multiple comparisons test (*HOXA10* and *β3* and Friedman test with post hoc Dunn’s multiple comparisons test for *SPP1* following tests for normality), *n* = 3 experiments.

To understand the mechanisms underlying the inhibition of model embryo–endometrial attachment by testosterone, the level of expression was measured in key genes known to be involved in receptivity of the human endometrium. Ishikawa cells were treated with 1 nM and 10 nM doses of testosterone, and the levels of *HOXA10*, integrin *β3* and *SPP1* (encoding osteopontin) were measured. The 10 nM dose of testosterone caused a significant decrease in all three studied genes when compared to the levels for untreated conditions (*p* = 0.008, *p* = 0.038, and *p* = 0.04 for *HOXA10*, *β3*, and *SPP1,* respectively; Figure 3A–C). No significant effect was measured in the expression of the three genes at the lower dose of 1 nM testosterone (*p* = 0.65, *p* = 0.11 and *p* = 0.99 for *HOXA10*, *β3* and *SPP1*, respectively, *n* = 3 experiments; Figure 3A–C).

**Figure 4:**
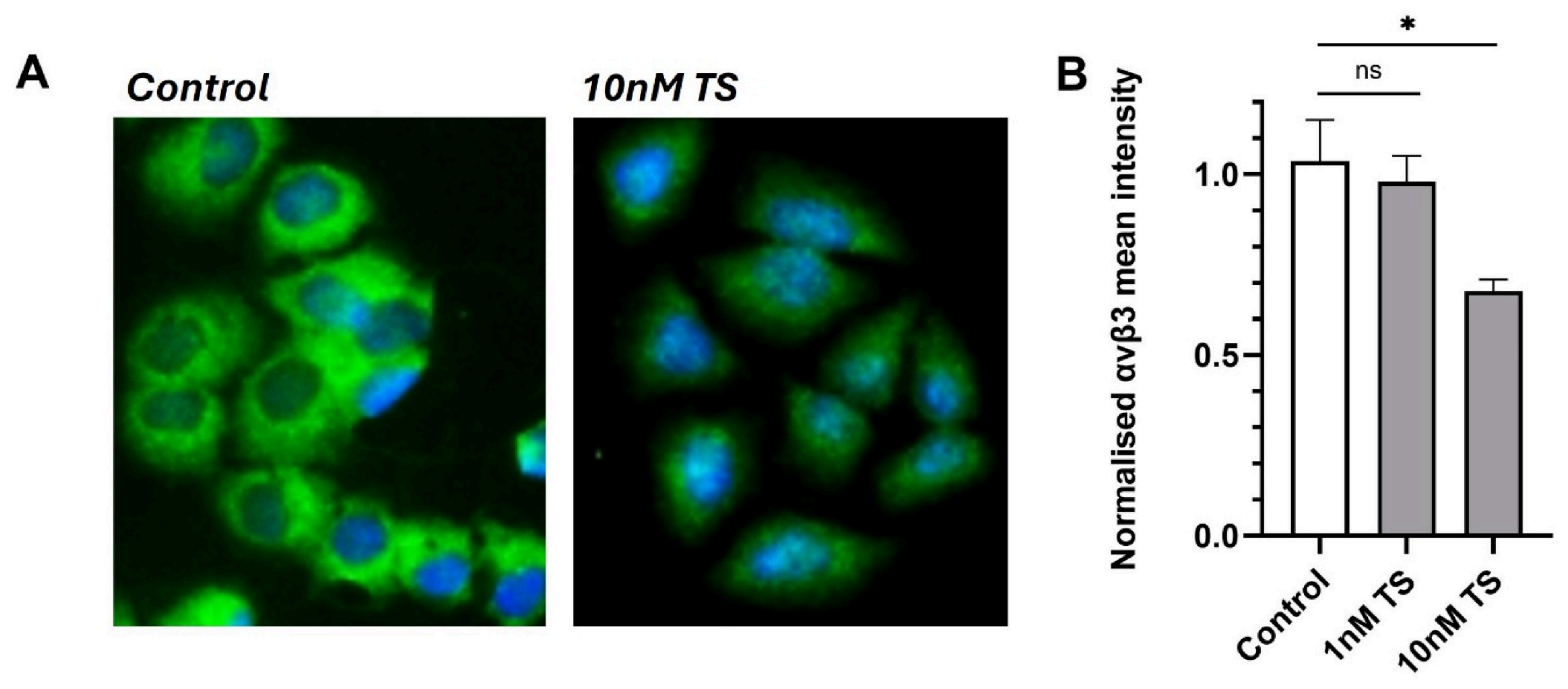
Expression of αvβ3 protein in Ishikawa cells following 24 h treatment with vehicle control, 1 nM or 10 nM testosterone (**A**). Green staining shows anti-mouse IgG-Alexa Fluor 488 αvβ3 protein, blue staining shows DAPI nuclear staining. Scale bar represents 25 µm. (**B**) Bar graph showing mean +/− SD of normalised mean fluorescence intensity from three experiments in the treatments described in (**A**). ns = not significant; * represents *p* < 0.05; ANOVA with post hoc Dunnett’s multiple comparisons test.

The significant effect of testosterone on *β3* gene expression was also measured at the protein level by immunocytochemistry, with 10 nM testosterone causing a significant decrease in αvβ3 protein expression following 24 h treatment of Ishikawa cells (*p* = 0.005; >100 analysed cells per treatment, *n* = 3 experiments; Figure 4A,B), although no significant effect on protein expression was determined at 1 nM (*p* = 0.93; Figure 4B).

### 3.4. Rescue of Embryo–Endometrial Attachment Using Flutamide

**Figure 5:**
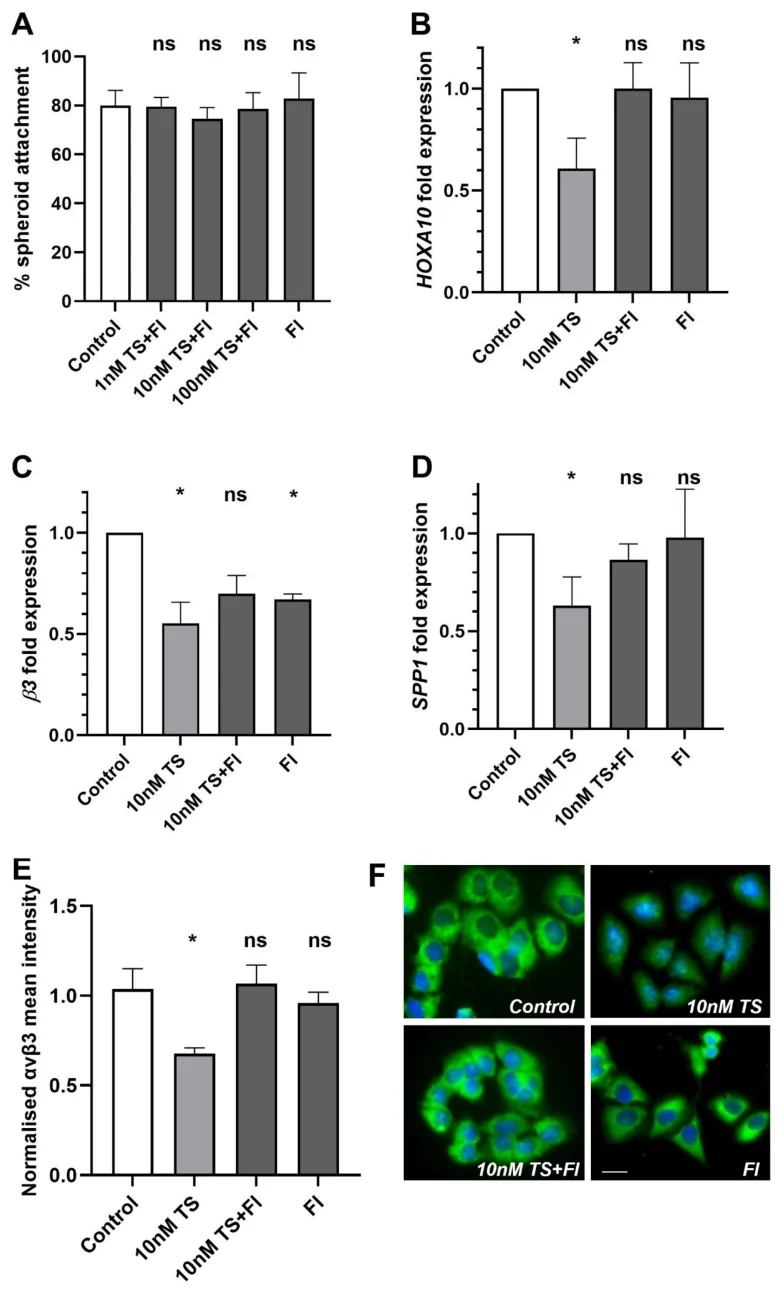
Effect of 1 µM flutamide treatment on testosterone-induced modifications in Ishikawa cells. (**A**) BeWo spheroid attachment to an Ishikawa cell monolayer pre-treated with 1 µM flutamide and increasing concentrations of testosterone (1 nM, 10 nM, 100 nM) for 24 h. Expression of (**B**) *HOXA10*, (**C**) *β3* and (**D**) *SPP1* genes, and (**E**) αvβ3 protein following 10 nM testosterone treatment alone, in combination with 1 µM flutamide, and with 1 µM flutamide alone. Graph shows normalised mean fluorescence intensity from three experiments. (**F**) Expression of αvβ3 detected using immunocytochemistry. Green staining shows anti-mouse IgG-Alexa Fluor 488 αvβ3 protein, blue staining shows DAPI nuclear staining. Scale bar represents 25 µm. Bar graphs show mean +/− SD. ns = not significant. * = *p* < 0.05; ANOVA with post hoc Dunnett’s multiple comparisons test, *n* = 3 experiments.

The nonsteroidal anti-androgen flutamide was used to determine whether it was possible to rescue the testosterone-induced decrease in model embryo–endometrial attachment and receptivity gene expression. Treatment with 1 µM flutamide rescued the decrease in testosterone-induced attachment at all testosterone concentrations (1 nM, 10 nM, and 100 nM, shown in Figure 2B), resulting in no significant difference in attachment levels compared to that of the control cells (*p* > 0.05 at all doses, *n* = 3; Figure 5A). No effect on cell viability was measured at this dose (96.9% viable compared to levels for the control; *p* = 0.202, *n* = 3 Supplementary Figure S1). Therefore, we set out to determine the mechanism by which flutamide exerted a positive effect on rescuing testosterone-inhibited model embryo–endometrial attachment. The effect of flutamide treatment on the expression of *HOXA10*, *β3* and *SPP1* in the presence and absence of 10 nM of testosterone was measured. The 10 nM testosterone-induced decrease in expression of all three genes was rescued with concurrent treatment with flutamide (Figure 5B–D). Interestingly, flutamide treatment alone had no significant effect on the expression level of *HOXA10* and *SPP1*, but significantly reduced the expression of *β3* compared to that of control cells (*p* = 0.005; *n* = 3; Figure 5C). This was not replicated at the protein level though, where flutamide was shown to rescue the 10 nM testosterone-induced decrease in the expression of αvβ3 protein (no significant difference between control and 10 nM TS + Fl treatment, *p* = 0.98; *n* = 3; Figure 5E,F). Flutamide had no significant effect on αvβ3 expression when added alone (*p* = 0.78; *n* = 3 experiments, >80 cells per treatment; Figure 5E,F).

## 4. Discussion

PMOS is a complex syndrome with a spectrum of symptoms, one of which is infertility. There are several characteristics of PMOS that could impact fertility, such as anovulation, insulin resistance and hyperandrogenism, the latter being the focus of the present study. An altered endometrial environment has been well-documented in PMOS, e.g., [33,34]. Testosterone has previously been shown to directly decrease the expression of HOXA10 in vitro, suggesting that the decreased endometrial receptivity seen in patients with PMOS could be, at least in part, due to the reduced HOXA10 expression [25,28]. A decrease in integrin αvβ3, important for embryo attachment, has also been observed during the window of implantation in women with PMOS [2,9].

This study aimed to investigate embryo attachment failure in PMOS using an in vitro model, thereby assessing the impact of testosterone on functional outcomes, as well as to evaluate key attachment proteins and their regulatory genes within the model. The influence of testosterone treatment on endometrial cells during embryo attachment was determined using spheroids generated from a choriocarcinoma trophoblast cell line to mimic the embryo. The effects of flutamide on the attachment of spheroids and the mechanisms of potential testosterone and flutamide action on implantation were also explored.

Endometrial cells were treated with a range of testosterone concentrations to mimic in vivo conditions, with 10 nM of testosterone chosen to represent pathological levels found in PMOS patients [35,36], along with the suprapathological dose of 100 nM. Whilst testosterone treatment did not affect endometrial cell viability nor morphology in the present study, it caused a significant decrease in spheroid attachment at elevated concentrations. This reduction in attachment is reflective of the decrease in successful embryo implantation rates in PMOS patients [29] and may be indicative of a reduction in endometrial receptivity. Whilst this has been previously proposed, this study is novel in its demonstration that attachment is indeed impaired under hyperandrogenic conditions in vitro.

To further investigate changes in receptivity, mRNA analysis of endometrial cells which had been exposed to physiological (1 nM) or elevated (10 nM) testosterone revealed downregulation of attachment-associated genes. A significant decrease in *HOXA10* expression was observed following 10 nM testosterone treatment. This finding is consistent with those of Cermik et al. [25] and corroborates the suggestion that hyperandrogenism may be responsible for the decreased expression of this transcription factor in the endometria of PMOS patients. The mechanism by which testosterone regulates *HOXA10* expression is at present unknown.

A dose-dependent reduction in *β3* integrin mRNA was detected, reaching significance following 10 nM testosterone treatment. As the β3 subunit has previously been shown to be directly regulated by HOXA10, this is an expected consequence of the concurrent reduction in the latter. Additionally, a dose-dependent decrease in *SPP1,* which encodes OPN, was observed, which also reached significance following 10 nM testosterone treatment. Whilst an association has previously been reported between OPN and testosterone treatment in the kidney [24,37], we are not aware of any investigation of the effects within the reproductive system. The implications of a reduction in this important αVβ3 ligand for embryo adhesion are substantial and would be expected to compound the deficit in the aforementioned integrin. A previous study that evaluated OPN in PMOS patients determined that levels were altered, with an increase in circulating OPN, but a decrease in endometrial expression [22]. Saklamaz et al. [23] proposed a defect in OPN-mediated signalling and that secretion of OPN is increased to circumvent the problem in PMOS patients. It is therefore possible that the in vitro testosterone exposure of cells undertaken in this study resulted in a reduction in endometrial OPN expression, which is reflective of the PMOS environment.

Analysis of the impact of the molecular changes on the production and localisation of αvβ3 by immunocytochemistry also revealed a downregulation of this protein following 10 nM testosterone treatment, though not for the 1 nM dose.

Flutamide, employed here as an androgen inhibitor, successfully rescued the decrease in spheroid attachment observed following testosterone treatment at each concentration tested, indicating that this treatment improves endometrial receptivity in hyperandrogenic conditions. This supports a previous study showing that flutamide improves endometrial receptivity by ameliorating decidualization and angiogenesis in testosterone-treated mice [38]. Additionally, we observed restoration of *HOXA10*, *β3* integrin subunit and *SPP1* expression to control levels following flutamide treatment, confirming the role of testosterone in the observed downregulation of these genes. αvβ3 protein production following testosterone treatment was also restored by exposure to flutamide, indicative of a potential mechanism for the improved levels of spheroid attachment described here. However, we observed that flutamide treatment alone caused a significant reduction in *β3* gene expression, though this was not evident at the αvβ3 protein level, thus suggesting that underlying complexity of this mechanism remains, requiring further elucidation. Although flutamide has been successfully employed as a treatment for hyperandrogenism in PMOS patients [30,39,40], its use is currently contraindicated in pregnancy [41]. Whilst useful here in aiding understanding of the impact of testosterone on the ability of embryo mimics to attach in vitro, an alternative therapeutic approach would potentially be required for modification of hyperandrogenism in human patients wishing to conceive.

The treatment regimens utilised in this study were representative of acute (24-h) exposure of the endometrium to testosterone and flutamide. However, chronic or prolonged treatment with these agents over several weeks of culture, as well as pre-treatment of the trophoblast spheroids, may provide an insight into the effects of longer-term chronic exposure, relevant to the PMOS environment. The blastocyst is itself known to produce αvβ3 [42], and therefore, it might be expected that exposure in utero to pathological levels of testosterone such as those found in PMOS patients would modify expression prior to implantation, potentially disrupting the implantation process further. These observations additionally highlight the need to regulate androgen levels in patients to optimise their likelihood of conception.

Although the use of trophoblast spheroids as an in vitro model provides an effective and accessible tool for the study of modification in attachment within a PMOS environment without the need for human or mouse embryo use, these are limited in their representation of the complexity of the signalling and adhesion characteristics of the human blastocyst in vivo. Future work could focus on the use of the recently reported assembloid [43] or blastoid models [44]. The attachment model could be adapted to incorporate additional features of PMOS, including insulin resistance, inflammation and susceptibility to environmental factors, as well as additional steroid hormone treatments in other endocrine disorders causing reduced fertility and for an extended duration beyond the 24 h examined in this model. Further work should also assess additional receptivity markers which have been reported as dysregulated in PMOS, including leukaemia inhibitory factor (LIF), miRNAs, the insulin-like growth factor (IGF) axis, and matrix metalloproteinases (MMPs). Whilst our findings are indicative of a testosterone-driven mode of action for attachment reduction, the specific mechanism of this modification is still to be elucidated.

## 5. Conclusions

Fundamentally, this in vitro study provides evidence to support the need for androgen regulation in the treatment of impaired endometrial receptivity and embryo attachment in PMOS. Future directions to address infertility in patients with PMOS should, in addition to the well-described ovulatory dysregulation, focus on improvements in endometrial receptivity to enhance pregnancy outcomes.

## Data Availability

Available on request.

## Supplementary Materials

The following supporting information can be downloaded at: https://www.mdpi.com/article/10.3390/cimb48101090/s1.

## Abbreviations

The following abbreviations are used in this manuscript:

PMOS	Polyendocrine Metabolic Ovarian Syndrome
PCOS	Polycystic Ovary Syndrome
IVF	In Vitro Fertilization
WOI	Window of Implantation
OPN	Osteopontin
LIF	Leukaemia Inhibitory Factor
IGF	Insulin-like Growth Factor
MMPs	Matrix Metalloproteinases
PVP	Polyvinylpyrrolidone
HOXA10	Homeobox A10
