# Supplementary material for: Testosterone Impairs Endometrial Receptivity Markers and Trophoblast Spheroid Attachment In Vitro: Reversal by Flutamide in a Model of Polyendocrine Metabolic Ovarian Syndrome
Source: Curr Issues Mol Biol. 2026 Oct 21;48(10):1090. doi: 10.3390/cimb48101090 (PMC13703127; doi:10.3390/cimb48101090)
Supplement: Supplementary file 1 [file cimb-48-01090-s001.zip › cimb-4585523-supplementary.pdf]

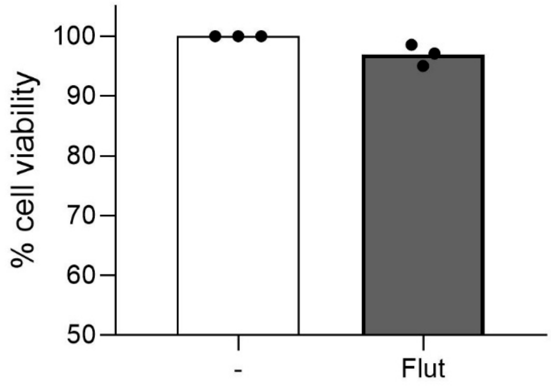

Figure S1 – Effect of 24h treatment with 1µM flutamide on the viability of Ishikawa cells. No significant difference compared to vehicle control (*p*=0.202, ANOVA with post hoc Dunnett's multiple comparisons test, *n* = 3 experiments.)
